# Structural understanding of SARS-CoV-2 virus entry to host cells

**DOI:** 10.3389/fmolb.2023.1288686

**Published:** 2023-11-02

**Authors:** Kim Le, Shrute Kannappan, Truc Kim, Jung Heon Lee, Hye-Ra Lee, Kyeong Kyu Kim

**Affiliations:** ^1^ Department of Precision Medicine, Sungkyunkwan University School of Medicine, Institute of Antibacterial Resistance Research and Therapeutics, Sungkyunkwan University, Suwon, Republic of Korea; ^2^ Research Center for Advanced Materials Technology Core Research Institute, Suwon, Republic of Korea; ^3^ School of Advanced Materials and Science Engineering, Sungkyunkwan University, Suwon, Republic of Korea; ^4^ Department of Biotechnology and Bioinformatics, College of Science and Technology, Korea University, Sejong, Republic of Korea

**Keywords:** cryo-EM, SARS-CoV-2, structure-guided drug design, virus entry, membrane fusion

## Abstract

Coronavirus disease 2019 (COVID-19), caused by the severe acute respiratory syndrome coronavirus 2 (SARS-CoV-2), is a major global health concern associated with millions of fatalities worldwide. Mutant variants of the virus have further exacerbated COVID-19 mortality and infection rates, emphasizing the urgent need for effective preventive strategies. Understanding the viral infection mechanism is crucial for developing therapeutics and vaccines. The entry of SARS-CoV-2 into host cells is a key step in the infection pathway and has been targeted for drug development. Despite numerous reviews of COVID-19 and the virus, there is a lack of comprehensive reviews focusing on the structural aspects of viral entry. In this review, we analyze structural changes in Spike proteins during the entry process, dividing the entry process into prebinding, receptor binding, proteolytic cleavage, and membrane fusion steps. By understanding the atomic-scale details of viral entry, we can better target the entry step for intervention strategies. We also examine the impacts of mutations in Spike proteins, including the Omicron variant, on viral entry. Structural information provides insights into the effects of mutations and can guide the development of therapeutics and vaccines. Finally, we discuss available structure-based approaches for the development of therapeutics and vaccines. Overall, this review provides a detailed analysis of the structural aspects of SARS-CoV-2 viral entry, highlighting its significance in the development of therapeutics and vaccines against COVID-19. Therefore, our review emphasizes the importance of structural information in combating SARS-CoV-2 infection.

## 1 Introduction

Coronavirus disease 2019 (COVID-19) pandemic, caused by severe acute respiratory syndrome coronavirus 2 (SARS-CoV-2), threatens the health of people worldwide. According to World Health Organization (WHO) statistics, as of August 2023, more than 769 million people have been infected worldwide, accounting for nearly 7 million deaths (WHO, 2023a). SARS-CoV-2 has four structural proteins, including the Spike protein (S), Membrane protein (M), Envelope protein (E), and Nucleocapsid protein (N), as well as several non-structural proteins, including ORF3a, ORF3b, ORF6, ORF7a, ORF7b, ORF8, ORF9b, ORF9c, and ORF10 (Wu et al., 2020b; Harrison et al., 2020; Michel et al., 2020). Among these, the Spike protein plays an important role in influencing viral pathogenesis by mediating viral entry through binding to host cells, thus inducing viral pathogenicity, and is essential for triggering host immune responses (Lu et al., 2015; Millet and Whittaker, 2015; Hulswit et al., 2016; Li, 2016).

Among human-infecting coronaviruses (CoVs) such as human coronavirus NL63, human coronavirus OC43, Middle East respiratory syndrome-related coronavirus (MERS), severe acute respiratory syndrome coronavirus (SARS), and SARS-CoV-2, only SARS-CoV-2 has caused a long-term-pandemic and widespread infections. This suggests that the molecular structure of the SARS-CoV-2 Spike protein differs from that of other human-infecting CoVs (Gaunt et al., 2010; Lau et al., 2011; WHO, 2023c; WHO, 2023d). Therefore, the Spike protein is key to understanding the SARS-CoV-2 and developing effective therapeutics. The current understanding of the viral entry mechanism involves the binding of the Spike protein of SARS-CoV-2 to the receptor on host cells, followed by the cleavage of Spike protein by the host protease and viral fusion to enter into the host cell (Shang et al., 2020a; Zamorano Cuervo and Grandvaux, 2020; Peng et al., 2021). In addition, like previous CoVs, both structural changes and exposure of protease cleavage site in the Spike protein are not only required for viral binding to the host cells but are critical for viral replication and thence are potential targets for therapeutic interventions (Bestle et al., 2020; Martinez-Flores et al., 2021). As of 30 March 2023, a total of 382 vaccine studies are under development, with 189 vaccines in clinical trials and 199 in preclinical trials (WHO, 2021). Among them, 32% (59 vaccines) are based on viral protein structure, the majority targeting the Spike protein (WHO, 2021). This supports the importance of studies that provide an understanding of the Spike protein structure.

Current structure-based studies using cryo-electron microscopy (cryo-EM) and X-ray crystallography have provided critical insights into the conformational changes and interactions of the SARS-CoV-2 Spike protein with host cells in different conformational states, including prebinding, binding to host cells, and postfusion states (Buchrieser et al., 2020; Cai et al., 2020; Wrapp et al., 2020; Zhang et al., 2021a; Cattin-Ortolá et al., 2021; Koppisetti et al., 2021). These structures have provided insights into the mechanism of viral fusion and the molecular details of conformational changes in the Spike protein. Structure-based research has also identified potential targets for the development of therapeutics, such as the receptor-binding motif (RBM) on the Spike protein, which is a primary target for neutralizing antibodies (Du et al., 2021; Kim et al., 2021).

Since the beginning of the pandemic in 2019, new variants have continuously emerged with concomitant increases in the level of infection (Bugembe et al., 2021; Grabowski et al., 2021; Kaleta et al., 2022). The WHO continued to announce variants of concern during the pandemic, including Alpha, Beta, Gamma, Delta, and Omicron variants, until March 2023 (WHO, 2022). During this time, the understanding of SARS-CoV-2 variants was gradually elucidated. The impact of viral mutations on aspects of SARS-CoV-2, such as transmission, virulence, host immune system evasion, vaccine effectiveness, diversity of antigenic interactions with antibodies, stability of protein, the flexibility of receptor-binding domain (RBD), and accessibility to human angiotensin-converting enzyme 2 (hACE2) receptor, are being studied (Kumar et al., 2023). Certain variants of SARS-CoV-2 have been shown to increase viral entry or virulence over others (Zhang et al., 2021c; Gupta et al., 2021; Harvey et al., 2021; Tao et al., 2021; Magazine et al., 2022). Thus, a structural perspective on these variants is of specific interest (Gupta et al., 2021; Harvey et al., 2021; Magazine et al., 2022). Many antiviral drugs that block the entry of viruses into cells have been developed due to the structure-based knowledge, which are now applied as vaccines and inhibitors of viral infection (Xia et al., 2020b; Choudhary et al., 2020; Xia et al., 2020c; Huang et al., 2020; Ou et al., 2020; Samrat et al., 2020; Sternberg and Naujokat, 2020; Mellott et al., 2021; Yang and Rao, 2021; Wang et al., 2022a).

Since the COVID-19 pandemic was first declared, many reviews have examined the SARS-CoV-2 infection mechanism and critical functions of various structural and non-structural proteins involved in this process, including receptor recognition, cleavage by host protease, and virus entry patterns into cells (Shang et al., 2020a; Haque et al., 2020; Huang et al., 2020; Kumavath et al., 2021; Jackson et al., 2022; Yan et al., 2022). However, to the best of our knowledge, none of them have provided an overview of the entry process mediated by Spike proteins and the effects of mutations on this process from a structural point of view (Harvey et al., 2021; Candido et al., 2022; Kim et al., 2022; McLean et al., 2022; Carabelli et al., 2023). Therefore, understanding the structural changes during each step of viral entry and the effects of mutations on protein structure, as well as Spike protein-protein interactions, is necessary to improve responses to SARS-CoV-2 and future strains of infectious CoVs.

This review aims to provide a detailed understanding of structural changes in the Spike protein of SARS-CoV-2 during entry into host cells. Its interactions with host proteins, membrane receptors, and proteolytic enzymes are also explained. We start with a section on the overall mechanism of viral entry into the host cells, followed by an in-depth discussion of each of the steps involved in virus entry from a structural biology perspective, classified here as prebinding, receptor binding, proteolytic cleavage, and membrane fusion steps. Although the entry process is generally divided into prefusion and postfusion stages, we divide this process into more defined steps for a clearer understanding. We also emphasize structural differences between the wild-type (WT) SARS-CoV-2 and its mutational variants at each step of the process. In addition, we explore the implications of structure-based approaches for the development of anti-viral therapeutics to combat COVID-19 in each step. In this review, we considered the Wuhan-Hu 1 strain as the WT virus, and thus, mutations in the Spike proteins mentioned in this review indicate the mutations on the Spike proteins of the Wuhan strain. In addition, we explain the furin cleavage process as the third step, even though this proteolytic cleavage occurs during virus maturation. The crystal and cryo-EM structures of the Spike protein used for generating the figures in this review are summarized in Table 1. In addition, all the small molecular inhibitors or therapeutic antibodies introduced in this review are listed in Table 2.

**TABLE 1:** Structures of SARS-CoV-2 Spike proteins and related proteins used in this review.

No.	PDB ID	Structure description	Figures	References
1	6VXX	WT Spike protein in closed conformation (three-RBD-down)	Figures 2, 3	Walls et al. (2020)
2	7R14	Alpha variant Spike protein in open conformation (one-RBD-up)	Figures 2, 5	Wrobel et al. (2022)
3	7VX1	Beta variant Spike in open conformation (one-RBD-up)	Figures 3, 5	Wang et al. (2021d)
4	7SBS	Gamma variant in open conformation (one-RBD-up)	Figure 5	Zhang et al. (2021b)
5	7W92	Delta variant in open conformation (one-RBD-up)	Figure 5	Wang et al. (2022c)
6	7TEI	Omicron variant in open conformation (one-RBD-up)	Figure 5	Gobeil et al. (2022)
7	7R17	Beta variant in open conformation (two-RBD-up)	Figures 2, 3	Wrobel et al. (2022)
8	7X7N	Spike-Δ19-D614G strain in open conformation (three-RBD-up)	Figures 2, 3	Khatri et al. (2022)
9	6M0J	WT Spike protein RBD domain in complex with hACE2	Figure 4	Lan et al. (2020)
10	7EKF	Alpha variant Spike protein RBD domain in complex with hACE2	Figure 4	Han et al. (2021)
11	7EKG	Beta variant Spike protein RBD domain in complex with hACE2	Figure 4	Han et al. (2021)
12	7EKC	Gamma variant Spike protein RBD domain in complex with hACE2	Figure 4	Han et al. (2021)
13	7TEW	Delta variant Spike protein RBD domain in complex with hACE2	Figure 4	Saville et al. (2022)
14	7U0N	Omicron variant Spike protein RBD domain in complex with hACE2	Figure 4	Geng et al. (2022)
15	6RXA	WT Spike protein in postfusion conformation	Figure 7	Cai et al. (2020)
16	6M1D	Full-length hACE2	Figure 2	Yan et al. (2020)
17	6YD4	Furin	Figure 6	Lam van et al. (2021)
18	7MEQ	TMPRSS2	Figures 2, 6	Fraser et al. (2022)
19	3K24	CTSL	Figures 2, 6	Adams-Cioaba et al. (2011)

Abbreviation: WT, Wild-type; RBD, receptor binding domain; hACE2, human Angiotensin-converting enzyme 2; TMPRSS2, Transmembrane protease serine 2; CTSL, cathepsin L.

**TABLE 2:** Representative inhibitors preventing the entry of SARS-CoV-2 into host cells in this review.

Step	Name	Type of molecule	Target	Mode of action	Notes	References
Prebinding	Nuvaxovid	Vaccine (protein)	hACE2	Full-length Spike protein, competitive with Spike protein in binding with hACE2	FDA emergency approval	Keech et al. (2020), Marchese et al. (2023)
	Covovax	Vaccine (protein)	hACE2	Full-length Spike protein, competitive with Spike protein in binding with hACE2	FDA emergency approval	Kanokudom et al. (2023)
	SKYCovione (GBP510)	Vaccine (protein)	hACE2	RBD Spike protein, competitive with RBD Spike protein in binding with hACE2	FDA emergency approval	Keech et al. (2020), Medicine (2023a)
	4A8	Neutralizing antibody	NTD	Inhibits Spike protein conformational changes	Activity confirmed in cell lines	Chi et al. (2020)
	COV2-2676	Neutralizing antibody	NTD	Inhibits postfusion steps	Activity confirmed in animal models	Suryadevara et al. (2021)
	COV2-2489	Neutralizing antibody	NTD	Inhibits postfusion steps	Activity confirmed in animal models	Suryadevara et al. (2021)
	COV2-3434	Neutralizing antibody	NTD	Disrupts Spike protein trimeric structure	Activity confirmed in animal models	Suryadevara et al. (2022)
Receptor binding	Dalbavancin	Glycopeptide	hACE2	hACE2 inhibitor	Activity confirmed in animal models	Wang et al. (2021b)
Proteolytic cleavage	Naphthofluorescein	Small molecule	Furin	Furin inhibitor	Activity confirmed in cell lines	Cheng et al. (2020)
	Decanoyl-RVKR-chloromethylketone	Small molecule	Furin	Furin inhibitor	Activity confirmed in cell lines	Cheng et al. (2020)
	Nafamostat mesylate	Small molecule	TMPRSS2	TMPRSS2 inhibitor	Phase 3 clinical trial	Tsukagoshi (2000), Hoffmann et al. (2020b), Hoffmann et al. (2020c), Mellott et al. (2021), Zhuravel et al. (2021)
	Camostat mesylate	Small molecule	TMPRSS2	TMPRSS2 inhibitor	Phase 2 clinical trial	Midgley et al. (1994), Hoffmann et al. (2020b), Gunst et al. (2021), Mellott et al. (2021)
	Amantadine	Small molecule	CTSL	CTSL Inhibitor	Phase 3 clinical trial	Fink et al. (2021), Zhao et al. (2021), Rejdak et al. (2022)
	K777	Small molecule	CTSL	CTSL Inhibitor	Phase 2 clinical trial	Mellott et al. (2021), Medicine (2023b)
Postfusion	EK1C4	Peptide	HR1	Inhibit HR1-HR2 formation	Activity confirmed in cell lines	Xia et al. (2020b)
	IPB02	Peptide	HR1	Inhibit HR1-HR2 formation	Activity confirmed in cell lines	Zhu et al. (2020)
	HR2P	Peptide	HR1	Inhibit HR1-HR2 formation	Activity confirmed in cell lines	Xia et al. (2020c)
	COV44-62	Neutralizing antibody	FP	Prevents FP binding to host cell membrane	Activity confirmed in cell lines	Dacon et al. (2022)
	COV44-79	Neutralizing antibody	FP	Prevents FP binding to host cell membrane	Activity confirmed in cell lines	Dacon et al. (2022)
	Salvianolic acid C	Natural compound	HR1	Fusion inhibitor	Activity confirmed in cell lines	Yang et al. (2020)

Abbreviation: HR1, Heptad repeat 1 domain; hACE2, human Angiotensin-converting enzyme 2; NTD, N-terminal domain; TMPRSS2, Transmembrane protease serine 2; CTSL, cathepsin L; FP, fusion peptide; FDA, US, food and drug administration; COVID-19, Coronavirus disease of 2019.

## 2 Overview of the SAR-CoV-2 Spike protein

### 2.1 WT Spike protein

SARS-CoV-2 is an enveloped, positive-sense, single-stranded RNA virus belonging to the family *Coronaviridae* in the order *Nidovirales* and in the group of β-CoVs (Paules et al., 2020). The outer surface of the virion is decorated with four structural proteins: Spike (S), Envelope (E), Membrane (M), and Nucleocapsid (N) proteins, out of which the Spike protein plays an important role in influencing viral pathogenesis by mediating interaction with the host cell membrane to induce viral pathogenesis via membrane fusion. Visually, the Spike protein forms a characteristic crown-like halo around the virion (Tang et al., 2020). Overall, the Spike protein is a type I membrane protein that consists of an extracellular N-terminal ectodomain anchored to the viral membrane by a transmembrane domain and a short cytoplasmic C-terminal domain. The Spike protein is a homotrimer, known as one of the largest class I fusion proteins with a weight of 423.6 kDa (without glycan) and ∼540 kDa (with glycan) (Rebelo et al., 2022). Structurally, it is a class I fusion protein (based on its secondary structure), which includes an α-helical trimer conformation that eventually folds into an α-helical hexamer (Schibli and Weissenhorn, 2004; Zhou et al., 2020). After the virus binds to the host cell receptor, the S1 subunit is detached, and the remaining Spike protein undergoes a conformational change to form an α-helical trimer that gains access to the host cell membrane. Simultaneously, another α-helical trimer, bound to viral membrane, folds into the α-helical trimer bound to the host cell membrane. This fusion of two α-helical trimers results in the formation of an α-helical hexamer or six-helix bundle (6-HB). The formation of 6-HB structure plays a crucial role in bringing the viral and host-membranes into close proximity, facilitating membrane fusion.

**FIGURE 1:**
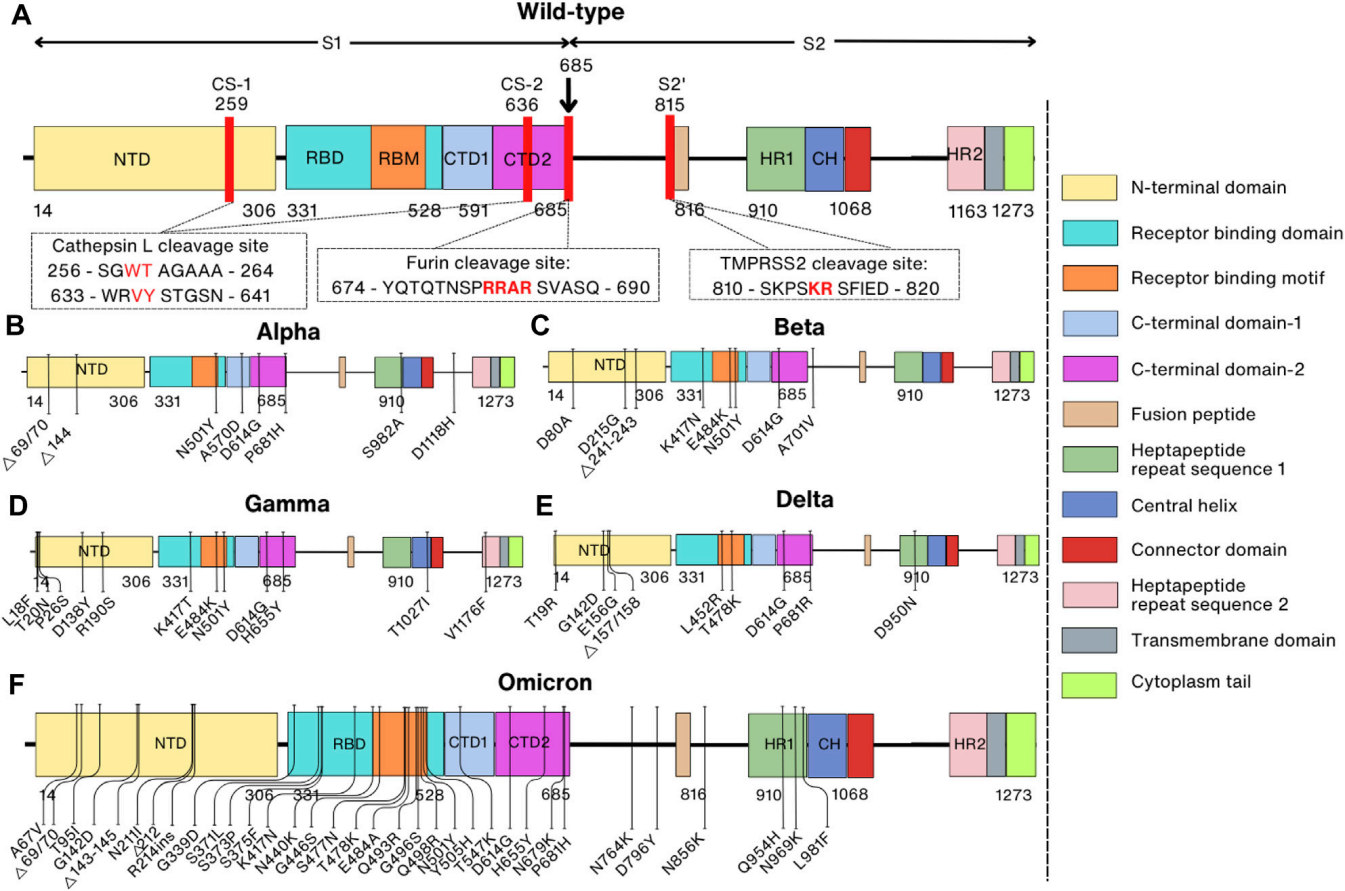
Comparison of domain representations between wild-type and mutant SARS-CoV-2 Spike proteins. Mutant Spike proteins from various variants are contrasted with the wild-type Spike protein: **(A)** Wild-type; **(B)** Alpha variant (B1.1.7); **(C)** Beta variant (B1.351); **(D)** Gamma variant (P1); **(E)** Delta variant (B.1.617.2); **(F)** Omicron variant (BA.1). The right panel specifies domain names and their corresponding colors. In the domain representation of the wild-type protein **(A)**, proteolytic cleavage sites are denoted by red lines, and the corresponding sequences are indicated within the box. Mutation sites in the mutant proteins are also depicted.

The Spike protein is composed of the S1 subunit (residues 1–685) and the S2 subunit (residues 686–1,273) (Hardenbrook and Zhang, 2022) structurally resembling a bulbous head and a stalk. The S1 subunit forms the globular head consisting of the N-terminal signal peptide (residues 1–13) followed by the N-terminal domain (NTD, residues 14–306), receptor-binding domain (RBD, residues 331–528) and the C-terminal domain (CTD1, residues 528–591, and CTD2, residues 592–685). The S2 subunit is shaped like a spicule rod and is made up of the fusion peptide (FP, residues 816–834), heptapeptide repeat sequence 1 (HR1, residues 910–985), central helix (CH, residues 986–1,035), connector domain (CD, residues 1,036–1,068), heptapeptide repeat sequence 2 (HR2, residues 1,163–1,211), transmembrane domain (TM, residues 1,212–1,234), and cytoplasm tail (CT, residues 1,235–1,273) (Figure 1A) (Duan et al., 2020; Zhang et al., 2021a). In its natural state, the Spike protein’s S1 and S2 domains are joined noncovalently by a furin cleavage site (Walls et al., 2020). In addition to the furin cleavage site, the SARS-CoV-2 Spike protein contains cleavage sites for two other proteases, cathepsin L (CTSL) and transmembrane serine protease 2 (TMPRSS2), which are important for the entry of SARS-CoV-2 into host cells (Figure 1A) (Zhao et al., 2022a; Essalmani et al., 2022).

Each component of the Spike protein has a specialized role in mediating the entry of SARS-CoV-2 into host cells. The NTD of the S1 subunit consists predominantly of β-sheets decorated with eight N-linked glycans that interact with host coreceptors and are involved in the formation of a stabilized protein structure as well as mounting immune response (Pierri, 2020; Berkowitz and Ostrov, 2022; Candido et al., 2022). The RBD in the S1 subunit is 220-residue-long, consisting of an extended loop named receptor binding motif (RBM) rich in polar residues and a core with nonpolar residues (Shang et al., 2020b; Lan et al., 2020). The RBD plays a vital role in binding to the hACE2 expressed on the surface of host cells, allowing the virus to undergo a conformational change to enter and infect the host cells. The CTD predominantly consists of β-sheets and is essential for the fusogenic structural rearrangements of the Spike protein (Zhang et al., 2021a). The FP in the S2 subunit is a short 19-amino acid segment composed mainly of hydrophobic residues such as glycine and alanine (Millet and Whittaker, 2018). It can structurally rearrange to expose the hydrophobic fusion loop of the FP for triggering membrane fusion upon contact with the cell surface receptor (Badani et al., 2014). The HR regions are also composed of conserved heptapeptide repeats of HPPHCPC (‘H’-hydrophobic, “P”-hydrophilic, and “C”-charged) (Chambers et al., 1990). The HR2 domain present in the N-terminus of the TM domain strongly binds to the HR1 domain located at the C-terminus of the FP and the mutual interaction between them forms a six-helix bundle (6-HB) that drives the membrane fusion (Cano-Muñoz et al., 2022; Pang et al., 2022).

Similar to other enveloped viruses, the SARS-CoV-2 Spike protein requires cell surface binding before its membrane is fused with the host cell membrane to begin an infection (Behzadipour et al., 2021). While the S1 subunits are responsible for viral infection by recognizing the host cell receptors, the S2 subunit is involved in the infection process by mediating the fusion of the virus with the host cell membrane. Spike protein also was proven to elicit a robust immune response (Nielsen et al., 2021). This feature makes it one of the primary targets for the development of vaccines against COVID-19.

### 2.2 Mutant Spike proteins

The whole genome mutation rate of SARS-CoV-2 is 6.677 × 10^−4^ substitutions per site per year, and the nucleotide mutation rate of the S gene is 8.066 × 10^−4^ substitutions per site per year (Wang et al., 2022b). The spontaneous mutation rate in the whole genome is 1.3 ± 0.2 × 10^−6^ per base per infection cycle. This is consistent with the Spike protein having undergone five times as many mutations as the corresponding genomic average due to positive selection (Amicone et al., 2022). The first SARS-CoV-2 variant was discovered in the United Kingdom in the late summer and early fall of 2020 (Andrew, 2020). The Pango lineage nomenclature system assigned this variety the classification of B.1.1.7, and the WHO later defined it as the Alpha variant (Volz et al., 2021). This and some later variants have been identified as variants of concern (VOCs), including Alpha (B.1.1.7), Beta (B.1.351), Gamma (P.1), Delta (B.1.617.2), and Omicron (BA.1, BA.2, BA.4/BA.5) (Jeong et al., 2022; WHO, 2022).

The Spike protein of the Alpha variant has a total of ten mutations, of which there are seven missense mutations (N501Y, A570D, P681H, D614G, T716I, S982A, and D1118H) and three deletions in residues H69, V70, and Y144 (Figure 1B) (Davies et al., 2021). These mutations were proven to enhance dynamic stability, conformational flexibility, and binding of furin to the SARS-CoV-2 Spike protein (Mohammad et al., 2021). At the same time, the Beta variant was initially identified in South Africa in October 2020 and also has ten mutations (D80A, D215G, Δ241–243, K417N, E484K, N501Y, D614G, and A701V) in the Spike protein that is speculated to increase the kinetics of receptor binding (faster binding) and viral fusion, thereby enhancing virus fitness (Figure 1C) (Wang et al., 2021d; Tegally et al., 2021). Over a short period, other variants of SARS-CoV-2 such as the Gamma variant in Brazil in December 2020, the Delta variant in India in February 2021, and the Omicron variant in South Africa on 24 November 2021 were also quickly reported (Figures 1D, E) (Faria et al., 2021; Joshi et al., 2022; Viana et al., 2022). While Gamma and Delta variants have only twelve and ten mutations in the Spike protein, respectively, the Omicron variant has 37 mutations compared to the prototype WT, and these mutations are mainly located in the RBD of the Spike protein (Figure 1F) (Tzou et al., 2022).

## 3 Overview of SARS-CoV-2 entry mechanism

**FIGURE 2:**
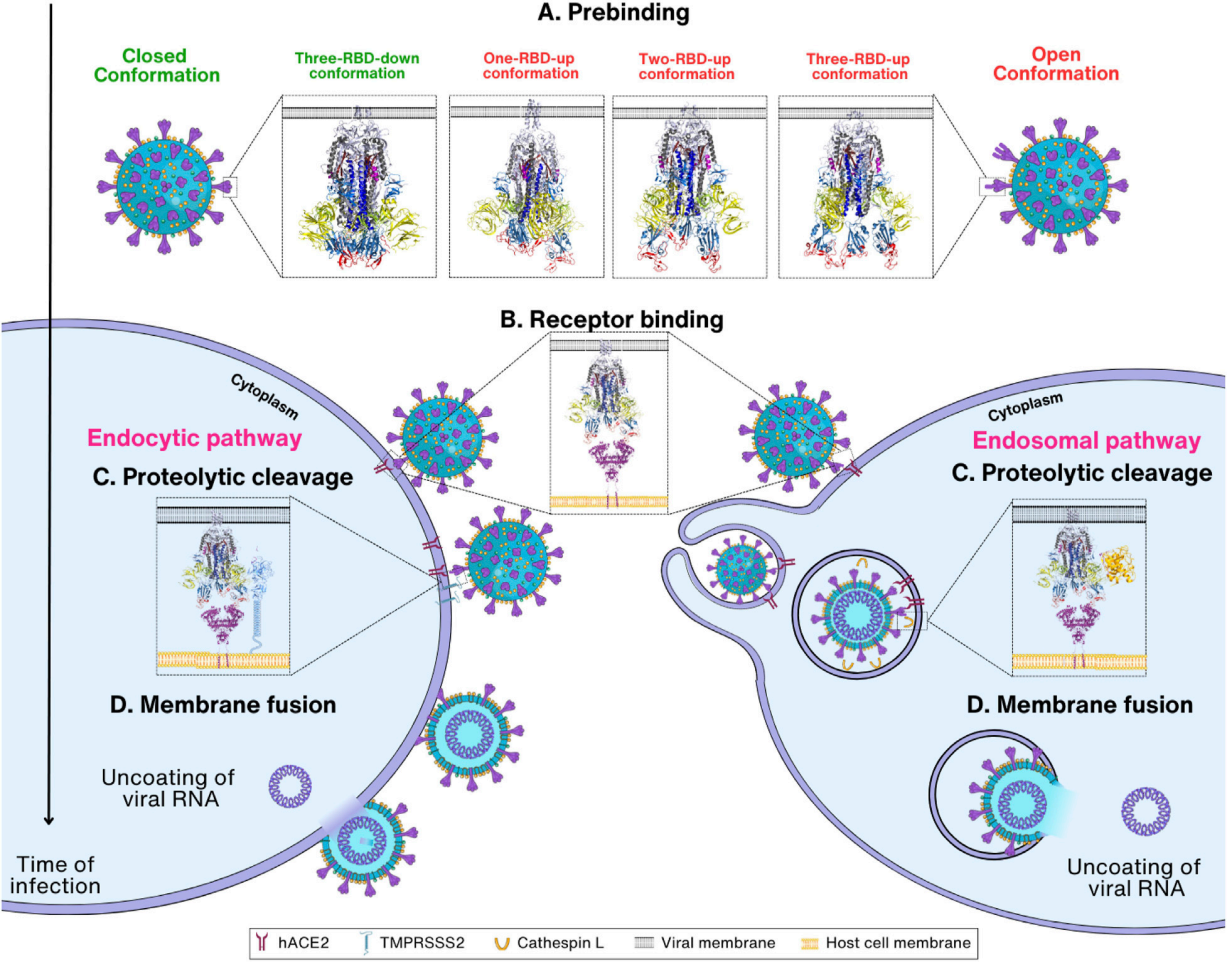
Overview of SARS-CoV-2 entry mechanism. **(A)** Prebinding step: Spike protein can be found in either the closed (PDB_ID: 6VXX) (Walls et al., 2020), or open conformation where one to three RBDs are exposed (PDB_ID:7VX1 (Wang et al., 2021d), 7R17 (Wrobel et al., 2022), and 7X7N (Khatri et al., 2022), respectively). **(B)** Receptor binding step: Spike protein in the open conformations interacts with the host cell receptor hACE2 through RBD (PDB_ID: 7R17 and 6M1D) (Yan et al., 2020; Wrobel et al., 2022). Depending on the TMPRSS2 concentration, the viral entry proceeds either via endocytic or endosomal pathways. **(C)** Proteolytic cleavage step: The S1 subunit is removed, followed by the cleavage of Spike protein by TMPRSS2 or cathepsin L, which results in the exposure of the S2 subunit. **(D)** Membrane fusion step: TMPRSS2 cleaves the Spike protein at S2′ site, removing the S1/S2′ segment and exposing the S2 subunit. Conformational change in S2 enables viral and host cell membrane fusion and further release of viral RNA inside cells.

The SARS-CoV-2 infection progresses through four steps: prebinding, receptor binding, proteolytic cleavage, and membrane fusion (Figure 2). Before the entry process, Spike proteins in the viral surface have a prebinding conformation that is ready for binding to the membrane receptors in the host cells. Attachment to the host cell by the Spike protein through receptor hACE2 on the host cell membrane is the second step for entry (Tortorici and Veesler, 2019). In the third step, the S2′ cleavage site of the Spike protein is cleaved by the TMPRSS2 present in the host cell membrane. However, for S2′ cleavage, the Spike protein must be primed by the cleavage of the S1/S2 boundary by furin during the virus maturation process. In the second step, virus can be transported into the host endosome via endocytosis when TMPRSS2 is absent in the host cell membranes. In this case, Spike protein is cleaved by CTSL in the endosome for membrane fusion. Eventually, in the membrane fusion step, FP and the transmembrane domain are brought together at the same end of the Spike protein due to a structural rearrangement, which ultimately leads to the insertion of the FP into the host membrane leading to membrane fusion (Hoffmann et al., 2020a) (Figure 2). However, it remains unclear whether the cleavage of CTSL at the non-S2′ region affects membrane fusion. Upon Spike protein cleavage by CTSL, a notable observation is that the Spike protein predominantly adopts an activated state (open/intermediate), as opposed to the untreated Spike, which remains in the closed state (Zhao et al., 2021). Remarkably, the FPPR region in the CTSL-treated Spike protein appears to be more dynamic in this state, suggesting that CTSL cleavage, particularly at the CS-2 site, could induce the exposure of the FP, facilitating membrane fusion. While experimental evidence has verified that CTSL cleavage at CS-1 and CS-2 is sufficient to promote membrane fusion, the detailed structural mechanisms have not yet been elucidated (Tang et al., 2020). The transient and nonspecific nature of CTSL may pose challenges in identifying cleavage sites within the Spike protein (Simmons et al., 2005).

In the first step, termed here the prebinding step, the Spike protein undergoes conformational changes with an exposed RBD, ready for the next step of binding to receptors. The conformation of the Spike protein in the prebinding step is important for the infection of the host by the virus (Pierri, 2020). The viral Spike protein is relatively unstable in its prebinding state and rapidly transits to an open conformation, which is necessary for recognizing and binding the host cells (Walls et al., 2020; Wrapp et al., 2020; Riley et al., 2021; Eilts et al., 2022) (Figure 2A). When the prebinding conformation has an exposed RBD, located on the surface of the Spike protein, it can mediate interactions with the receptor on the surface of the host cell. The interaction between the Spike protein and the receptor is required for virus entry into the host cell by allowing proteolytic cleavage followed by membrane fusion (Lan et al., 2020; Tai et al., 2020; Jawad et al., 2021) (Figure 2B). SARS-CoV-2 binds to the same cell entrance receptor as SARS-CoV, hACE2 (Zhou et al., 2020). In addition to hACE2, CD147, heparan sulfate, and Neuropilin-1 have also been identified as receptors for entry of SARS-CoV-2 into host cells (Cantuti-Castelvetri et al., 2020; Behl et al., 2022; Eilts et al., 2022). However, no complex structures have yet been reported for these receptors except hACE2, and thus this review will focus on the structural changes of Spike proteins upon entry into host cells using only hACE2 as the sole entry receptors. After binding to the host cell receptor, Spike protein is cleaved by proteases in the host cells including TMPRSS2 and CTSL (Xia et al., 2020a; Zhao et al., 2022a; Essalmani et al., 2022) (Figure 2C). This step is extremely important as it induces a large conformational change, revealing the S2 subunit followed by the shedding of S1 and activation of drastic S2 refolding into a postfusion state (Almehdi et al., 2021) (Figure 2D)*.* After the membrane fusion stage, when the cell membranes of the virus and host cell are fused, the virus releases its genetic material and initiates other infectious processes (Zhang et al., 2021d; Pizzato et al., 2022). With this, the role of the Spike protein is considered complete in the host cell infection of SARS-CoV-2.

## 4 Prebinding step

### 4.1 Prebinding conformation of the WT Spike protein

In the prebinding step, the Spike protein is in a state that is poised to undergo a conformational change upon binding to the host cell receptor (Walls et al., 2020). Spike protein is cleaved into S1 and S2 subunits after cleavage by the host proprotein convertase furin at the S1/S2 cleavage site (the so-called furin cleavage site) (Walls et al., 2020). The S1 subunit (residues 14–685) of the Spike protein containing the RBD and NTD in a V-shaped configuration plays a role in recognizing and binding to the host cell receptors (Peters et al., 2021), while the S2 subunit (residue 686–1,273) containing FP, CH, CD, HR1, HR2, TM, and CT is responsible for membrane fusion to the host cells (Figure 1A).

**FIGURE 3:**
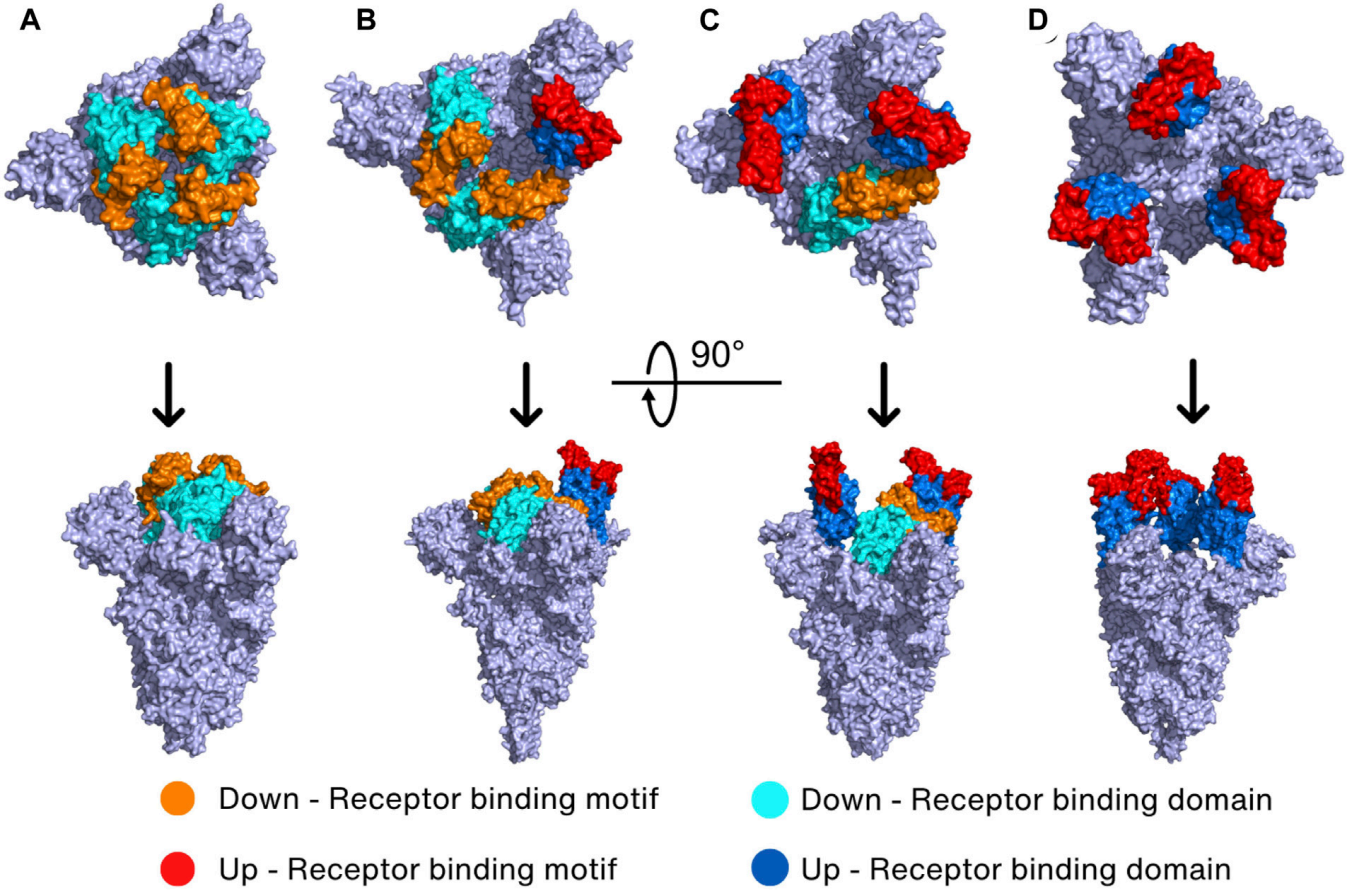
Prebinding conformation of the Spike protein in two different orientations. The Spike protein of SARS-CoV-2 exhibits four prebinding conformations with variations in the up-and-down position of the RBD: **(A)** closed conformation (PDB_ID: 6VXX) (Walls et al., 2020) where all RBDs are down; **(B)** one-RBD-up conformation (PDB_ID: 7VX1) (Wang et al., 2021d); **(C)** two-RBD-up conformation (PDB_ID: 7R17) (Wrobel et al., 2022); **(D)** three-RBD-up conformation (PDB_ID: 7X7N) (Khatri et al., 2022). The color of the down RBD, down RBM, up RBD, and up RBM are cyan, orange, marine, and red, respectively.

The Spike protein has four different conformations in its prebinding state depending on the position of RBD in the trimeric Spike protein. The hACE2-inaccessible state is the closed conformation in which RBD is found in the three-RBD-down position (Figure 3A). The one-RBD-up, two-RBD-up, and three-RBD-up conformations are open conformations, and thus hACE2 can access the exposed RBDs when Spike protein has the open conformation (He et al., 2020; Wan et al., 2020; Yi et al., 2020) (Figures 3B–D). Especially, the open conformation reveals a receptor-binding motif (RBM, S438–Y508), a short peptide located in the RBD of the S1 subunit that is responsible for binding to the host receptor. RBM consists of a loop structure that protrudes from the surface of the Spike protein, which enables RBM interaction with the host cell receptors in a highly specific manner (Lan et al., 2020).

It has been reported that the WT Spike protein has only two conformations including closed conformation and one-RBD-up conformation (Walls et al., 2020; Yurkovetskiy et al., 2020). Molecular dynamics (MD) simulation and cryo-EM studies revealed that closed conformation creates many interdomain salt bridges and hydrogen bonds, suggesting that closed conformation shows less mobility than the one-RBD-up conformation (Gur et al., 2020; Wang et al., 2021d). With higher mobility, the open one-RBD-up conformation can induce easier conformation changes, potentially enhancing viral infectivity. It is interesting to note that a semi-open intermediate conformation contains one RBD in a halfway position between down and up positions, and two other RBDs in a down position (Gur et al., 2020). This “semi-open intermediate conformation” seems to be a transition state between closed to open conformation, necessary for reducing the amount of energy needed to cross the energy barrier (Gur et al., 2020).

### 4.2 Effects of mutations on the prebinding conformation

Mutations in the Spike protein cause several conformational changes, playing a significant role in enhancing SARS-CoV-2 infectivity. Specifically, mutants that evolved at a later time point such as Omicron were more likely to have open conformation of the Spike protein. The distribution of the open and closed conformation of RBD in the WT strain is quite uniform with 53% in the close conformation and 47% in the one-RBD-up conformation (Wrapp et al., 2020; Yurkovetskiy et al., 2020). However, in variants with the D614G mutation, the rates of one- and two-RBD-up conformation were found to be 36% and 39%, respectively. Surprisingly, 20% of Spike proteins were present in an all-three-RBD-up conformations, and only 5% in the closed state in the D614G mutant (Yurkovetskiy et al., 2020). Moreover, the Omicron variant’s Spike protein has a 100% one-RBD-up conformation (Ye et al., 2022), explaining the highest infection phenotype of the Omicron variant.

The high prevalence of the open conformation in variants can be explained by the role of each mutation site. For example, D614 was identified as a critical glue point in the inter-protomer stabilization of the Spike protein (Peters et al., 2021), which explains why the D614G mutant has a 95% open conformation. In the Alpha variant, the combination of D614G and N501Y helps it gain a more open conformation. While D614G disrupts the salt bridge between D614 and K854 in the S2 subunit, the N501Y mutation induces hydrophobic interactions among Y501, V483, and F486 (Cai et al., 2020; Valério et al., 2022; Xiong et al., 2020). These hydrophobic interactions prevent the formation of salt bridges between E484 and R403, which is necessary for the stability of the closed conformation (Valério et al., 2022). Besides, the K417N mutation, which is additionally found in the Beta variant, also stabilizes the open conformation of the Spike protein by disrupting a salt bridge between K417 and E484 that stabilizes the closed conformation (Valério et al., 2022). In the Omicron variant, S371L, S373P, and S375F, located in the hairpin loop (residues 369–379) of RBD, also enhance the stability of the open conformation, supporting the highest prevalence of this conformation (Zhao et al., 2022b). Since these hydrophilic residues are involved in intramolecular interactions in the WT Spike protein, these mutations to hydrophobic residues in the Omicron Spike proteins are likely to affect the closed conformation (Cui et al., 2022; Lan et al., 2022).

### 4.3 Viral infection intervention by targeting the prebinding conformation of the Spike protein

The S1 subunit is the immunodominant antigen during SARS-CoV-2 infection since it is accessible for immune recognition and contains neutralizing epitopes on RBD. Therefore, a prebinding conformation is desirable for vaccine development. However, the prebinding conformation is metastable and thus prone to change the conformation in the next step, resulting in shedding the S1 subunit (Lee et al., 2022). Therefore, mutations in the WT Spike protein are strategically introduced to incorporate proline mutations, which serve to enhance the protein expression and stability. The same strategy was employed for developing vaccines against COVID-19 (Hsieh et al., 2020; Lu et al., 2022) and a variant named HexaPro with Pro mutations in six residues (Hsieh et al., 2020). Hexapro have shown enhanced efficiency in inducing antibodies to neutralize SARS-CoV-2 variants compared to the WT strain (Lu et al., 2022). Hexapro contains K986P and K987P in the loop between HR1 and the central helix. The corresponding mutations in MERS-CoV are reported to play a critical role in preventing premature triggering of the exposed state of the fusion protein (Pallesen et al., 2017). Furthermore, the presence of the F817P mutation in the FP and A892P and A899P mutations in the loop region connecting the FP and HR1 contributes to loop stabilization in Hexapro (Hsieh et al., 2020). The A942P mutation in the loop between the connector region and HR1 also contributes to stability by imposing the rigidity.

SARS-CoV-2 vaccines using the Spike protein as a critical component have been developed by various companies and are in use worldwide, with some still under further development or investigation. According to WHO’s March 2023 data, 59 vaccines utilizing the Spike protein are in clinical trials (WHO, 2021), of which 13 have WHO emergency use approval during the COVID-19 pandemic (WHO, 2023b). Among them, three vaccines incorporate the Spike protein or its subunit/domain: Nuvaxovid vaccine (Novavax) and Covovax vaccine (Serum Institute of India Pvt. Ltd.) employ recombinant full-length Spike protein (Kanokudom et al., 2023; Marchese et al., 2023), and SKYCovione (GBP510, SK bioscience) uses RBD-presenting nanoparticles (Keech et al., 2020; Medicine, 2023a). Moreover, research highlights neutralizing antibodies (nAbs) targeting the NTD, exhibiting a robust neutralization (Chi et al., 2020; Du et al., 2021; Li et al., 2023). Notably, 4A8 nAb does not impede hACE2 interaction but inhibits Spike protein conformational changes, hindering further infectious steps (Chi et al., 2020; Du et al., 2021; Li et al., 2023). COV2-2676 and COV2-2489 nAbs prevent SARS-CoV-2 infection effectively, without affecting RBD-hACE2 interaction as confirmed in cell lines and mouse models (Suryadevara et al., 2021). COV2-3434 disrupts Spike protein trimer structure by binding residues in NTD, rendering protection in mice against infection and promoting weight loss when administered prophylactically (Suryadevara et al., 2022).

## 5 Receptor binding step

### 5.1 Receptor binding conformation of the WT Spike protein

**FIGURE 4:**
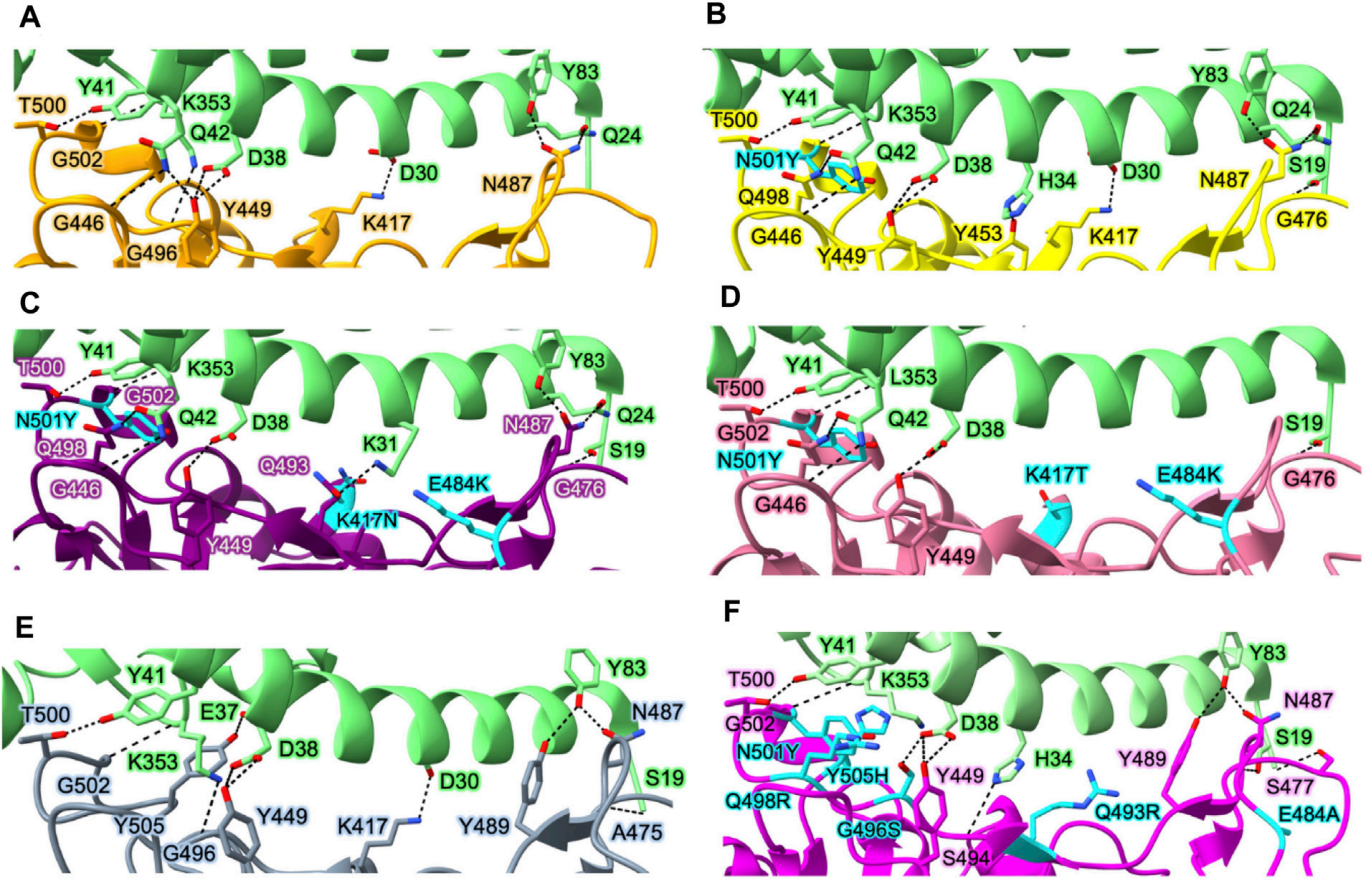
Interactions between the Spike protein and hACE2 in various variants. Essential residues involved in interactions between hACE2 and WT Spike protein **(A)**, Alpha variant **(B)**, Beta variant **(C)**, Gamma variant **(D)**, Delta variant **(E)**, and Omicron variant **(F)**. Protein structures are represented as ribbons, while the side chains of interacting residues are displayed as sticks (green, hACE2; orange, WT Spike protein; yellow, Alpha variant; purple, Beta variant; gray, Delta variant; pink, Omicron variant). Key mutation residues affecting the binding affinity between the Spike protein and hACE2 in each variant are highlighted in cyan. Protein structures are collected from PDB for hACE2 in complex with WT Spike protein (PDB_ID: 6M0J) (Lan et al., 2020), Alpha variant (PDB_ID: 7EKF) (Han et al., 2021), Beta variant (PDB_ID: 7EKG) (Han et al., 2021), Gamma variant (PDB_ID: 7EKC) (Han et al., 2021), Delta variant (PDB_ID: 7TEW) (Saville et al., 2022), and Omicron variant (PDB_ID: 7U0N) (Geng et al., 2022).

SARS-CoV-2 has a higher infectivity than SARS-CoV, even though their genomes share a 76% sequence overlap (Chan et al., 2020). While the binding of the viral Spike protein to the hACE2 of the host cell is a common entrance mechanism of both SARS-CoV and SARS-CoV-2, the difference between the two lies in the conformation of the Spike protein (Hoffmann et al., 2020b; Zhang et al., 2020; Zhou et al., 2020). Since SARS-CoV-2 has a higher fraction of Spike proteins in the “RBD-up” conformation, this translates to greater infectivity of SARS-CoV-2 in host cells by mediating stable binding to the hACE2 receptor. The formation of hydrogen bonds and salt bridges between several polar residues in the RBD and hACE2 throughout this process primarily governs RBD-hACE2 interactions (Figure 4A). The key interactions include T500–K41, N487–Y83, N487–Q24, and K417–D30 (residue numbers in virus and host order).

Upon hACE2 binding, the Spike protein in one- or two-RBD-up conformation can conformationally shift towards the three-RBD-up state (Yang et al., 2022b). The single-molecule Förster resonance energy transfer (smFRET) studies also reveal that hACE2 enhances the shift of the conformation population of Spike protein to a three-RBD-up conformation (Yang et al., 2022b). Hydrogen/deuterium-exchange mass spectrometry has revealed that RBM is the main binding site to hACE2 based on the decreased exchange rate in RBM after hACE2 binding (Chen et al., 2023a). Interestingly, an allosteric conformational change was also observed at the N-terminal region of the S2 subunit upon receptor binding, thereby exposing the S2′ cleavage site and facilitating the accessibility of TMPRSS2 (Chen et al., 2023a).

### 5.2 Effect of mutations on receptor binding conformation of the Spike protein

The affinities of the Spike proteins of Alpha, Beta, and Gamma variants to hACE2 are stronger than those of the WT Spike protein. In Alpha variant, N501Y induces a new π-π interaction between hACE2 and Spike resulting in a 10-fold increase in affinity between hACE2 and the RBD of Spike with respect to the WT (Yang et al., 2021; Liu et al., 2022) (Figure 4B). Although there are 10 mutations in the Spike protein of Alpha variant, only N501Y in the RBM region is involved in the RBD-hACE2 interaction. Beta and Gamma variants commonly have mutations at K417, E484, and N501 in the Spike protein involved in the interaction with hACE2. Both Beta and Gamma RBDs showed slightly lower binding affinities for hACE2 than Alpha RBD despite having two more mutated residues in the RBD domain (Wang et al., 2021d; Han et al., 2021). In particular, since K417 forms a tight contact with D30 of hACE2 via salt bridge between the positively charged side chain of K417 and the negatively charged side chain of D30, K417N reduces the affinity of RBD-hACE2 five-fold due to the loss of the salt bridge (Barton et al., 2021; Liu et al., 2021b; Liu et al., 2022). A weak salt bridge is broken by the E484K mutation since E484 in the WT virus’s Spike protein forms a weak salt bridge with K31 of hACE2 (Figures 4C, D). However, both Beta and Gamma variants still have higher affinity than the WT strain due to the presence of N501Y. Delta variant lacks the N501Y mutation (Figure 4E) but carry the L452R and T478K mutations. Therefore, the combination of two mutations, L452R and T478K, results in increasing the affinity of RBD to hACE2 compared to the WT Spike protein (Liu et al., 2022). The L452R mutation abrogates a hydrophobic patch composed of amino acids L452, L492, and F490, leading to the loss of stability of RBD and its ability to form a complex with hACE2 (Cherian et al., 2021). Although the T478 does not directly interact with hACE2, it has been observed to slightly enhance the affinity between hACE2 and RBD due to the formation of a hydrogen bond between the K478 and Q24 of hACE2 (Lan et al., 2020; Liu et al., 2021a).

In the Omicron variant, RBD contains 10 mutations on the hACE2 binding interface (N440K, G446S, S477N, T478K, E484A, Q493R, G496S, Q498R, N501Y, and Y505H) (Figure 4F). These mutations are speculated to impact the ability of the virus to recognize specific host cell receptors, and thus affect the entry of viruses into host cells (Cui et al., 2022). In particular, the mutation S477N forms a new interaction with S19 and Q24 in hACE2. The mutations N478K, Q493K, and Q498R make Omicron RBD more electrostatically positive, which contributes to the increased binding affinity to hACE2 (Niu et al., 2021). G496S, Q498R, and N501Y mutations also result in forming new interactions between the Omicron RBD and hACE2: hydrogen bond between Omicron’s S496 and hACE2’s D38, both hydrogen bond and salt bridge between Omicron’s R498 and hACE2’s Q42, and hydrophobic interaction between Omicron’s Y501 and hACE2’s K535 (Lan et al., 2022). While the NTD mutations are situated in the flexible loops distal from the trimer axis, the RBD mutations are primarily grouped close to the inter-protomer RBD-RBD interface, and several coincide with the hACE2-binding region.

Consistent with the structural studies, computational methods such as MD simulations in combination with molecular mechanics generalized Born surface area (MM/GBSA) and experimental techniques such as surface plasmon resonance (SPR) demonstrated that the Omicron variant has a stronger binding to hACE2 compared to other variants (Cui et al., 2022; Lan et al., 2022). This is particularly because of the mutation of the polar residues to basic residues in N440K, T478K, Q493K, and Q498R. Additionally, the acidic residue E484 is also lost when mutated to A484. These mutations result in a strong positive electrostatic potential at the surface of the RBD in Omicron, leading to high affinity binding to hACE2 (Hu et al., 2022).

### 5.3 Viral infection intervention by targeting the Spike protein-receptor interaction

With the development of structure determination techniques such as X-ray crystallography, nuclear magnetic resonance (NMR) spectroscopy, and cryo-EM, the structure of the whole virus, viral Spike protein, Spike protein-antibody, and Spike protein-human receptor complex can be determined at near-atomic resolution (Wang et al., 2022d; Li et al., 2022; Ozawa et al., 2022; Zhan et al., 2022). Based on that advantage, many treatments targeting the Spike protein and its receptors have been researched and developed, mainly focusing on targeting RBD domain of the Spike protein or hACE2 to prevent their interaction (Huo et al., 2020; Tai et al., 2020; Otsubo et al., 2022).

Antibodies are notably developed just after vaccine development and can neutralize SARS-CoV-2. These antibodies mainly target the RBD region. There are two types of antibodies useful for treating COVID-19: nAbs and monoclonal antibodies (mAbs) (Brobst and Borger, 2021; Hwang et al., 2022). The sources of nAbs and mAbs are different. While nAbs can be naturally produced by the immune systems of individuals who have recovered from COVID-19 or after convalescent plasma therapy, mAbs are developed in the laboratory through biotechnology techniques. mAbs are designed to be identical copies of antibodies that specifically target a particular epitope on the SARS-CoV-2 Spike protein (Jiang et al., 2020; Hwang et al., 2022). Due to the ability of the antibodies to compete with hACE2 for binding to RBD and their ability to selectively bind to specific conformation of the RBD (up/down conformation), the nAbs/mAbs are divided into four main categories (Barnes et al., 2020a). Class 1 antibodies compete directly with hACE2 for binding to RBD and only bind to RBD in the up conformation, class 2 antibodies also compete directly with hACE2 but bind to RBD in both the up and down conformations, class 3 antibodies do not compete with the hACE2 binding site and recognize both the up and down conformations of the RBD, and class 4 antibodies also do not compete with the hACE2 binding site but recognize only the RBD in the up conformation (Barnes et al., 2020a; Shrestha et al., 2021; Chen et al., 2023b). In convalescent and vaccinated individuals, RBD is the primary target of serum-neutralizing activity as it contains numerous antigenic sites identified by nAbs with varying neutralization potencies and breadth (Barnes et al., 2020b; Piccoli et al., 2020; Pinto et al., 2020; Greaney et al., 2021; Starr et al., 2021; Tortorici et al., 2021). Since many nAbs detect the two RBD-up positions, in order to boost neutralization, a lower number of RBD-up positions may cause this effect to be less pronounced due to lower avidity (Liu et al., 2020a; Rapp et al., 2021).

**FIGURE 5:**
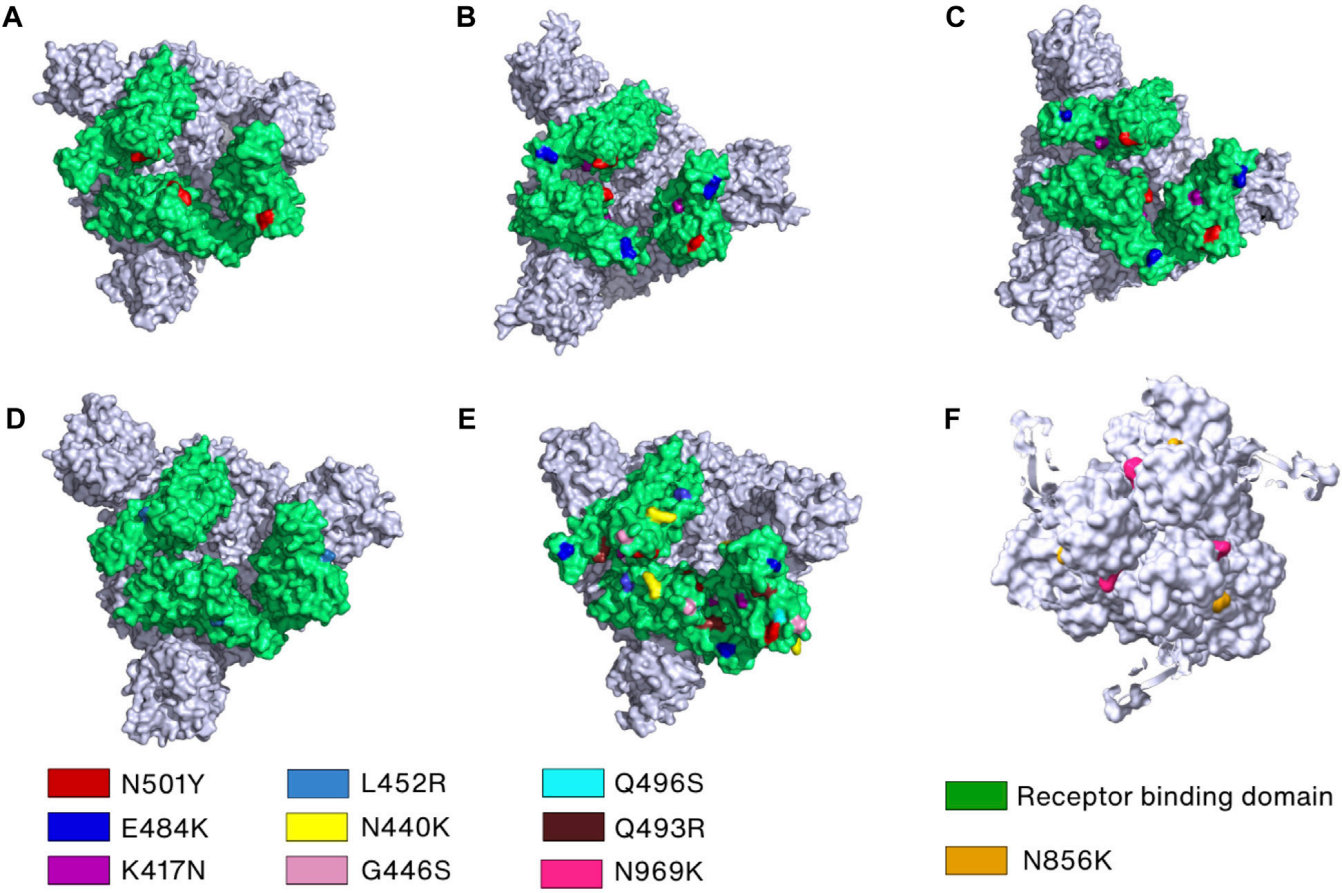
Key mutations in the Spike protein of the SARS-CoV-2 variants. **(A–E)** The crucial mutation sites in the RBDs of Spike protein of the Alpha (PDB_ID: 7R14) (Wrobel et al., 2022), Beta (PDB_ID: 7VX1) (Wang et al., 2021d), Gamma (PDB_ID: 7SBS) (Zhang et al., 2021b), Delta (PDB_ID: 7W92) (Wang et al., 2022c), and Omicron variants (PDB_ID: 7TEI) (Gobeil et al., 2022), respectively, are marked by different colors and labels or labelled on the surface filling models of the Spike proteins. **(F)** Mutations in the S2 subunit of the Omicron variant are indicated and labeled.

Mutations also play an important role in SARS-CoV-2 immune evasion. In the Alpha variant, N501Y and ΔY144 (deletion mutant) have been reported to increase the immune escape of SARS-CoV-2 since N501 and Y144, located in RBD and NTD, respectively, are the major antibody binding sites (Figure 5A) (Li et al., 2021a; McCallum et al., 2021; McCarthy et al., 2021; Kudriavtsev et al., 2022). The N501Y mutation site is exposed in RBD in the up conformation but buried in the RBD-down conformation. In addition to N501Y, two more mutations were found in Beta variant (K417N and E484K) and Gamma variant (K417T and E484K) that can reduce susceptibility of several mAbs (Baum et al., 2020; Chen et al., 2021; Copin et al., 2021; Wang et al., 2021c) (Figures 5B, C). Additionally, only the L452R mutation in Delta variant is linked to significant resistance levels and decreased sensitivity to several mAbs, including bamlanivimab (Li et al., 2020; Copin et al., 2021; Liu et al., 2021d) (Figure 5D). The cryo-EM structure revealed that neutralization against the Omicron Spike protein is impaired by steric collision and diminished polar contacts (Cerutti et al., 2022) caused by the following mutations: S371L, K417N, N440K, G446S, E484A, Q493R, G496S, N501Y, N856K, and N969K (Tzou et al., 2022) (Figures 5E, F).

Besides Spike proteins, hACE2 is also a target for developing inhibitors that can prevent infection by SARS-CoV-2. Although there is no evidence from patient studies, dalbavancin, a lipoglycopeptide antibiotic, has been shown to inhibit the replication of SARS-CoV-2 and pathogenicity in mouse and rhesus macaque models (Wang et al., 2021b). This inhibition occurs because dalbavancin binds to hACE2 at residues E329, Q325, Q42, and D38 in the hACE2-Spike protein interface, competing with RBM of Spike protein, thereby preventing those interactions (Wang et al., 2021b).

## 6 Proteolytic cleavage step

### 6.1 Proteolytic cleavage of the WT Spike protein

Furin has been discovered to be an integral protease for priming the fusion of surface glycoproteins of a variety of viruses in the extensive pH range of 5–8 (Thomas, 2002; Garten, 2018). Phylogenetic analysis revealed that SARS-CoV-2 Spike protein has a furin cleavage motif (682-RRAR-685) that can be recognized by furin, but this site is not present in the Spike proteins of other SARS-CoVs with 96% genomic sequence similarity to SARS-CoV-2 (Xia et al., 2020a; Coutard et al., 2020). This unique feature, combined with the rapid transmission and wide impact of SARS-CoV-2 compared with previous CoVs, emphasizes the importance of furin cleavage in SARS-CoV-2 pathogenesis.

Based on structural, functional, and biophysical studies, it was determined that three furin proteases bind S1 subunits of the SARS-CoV-2 Spike protein homotrimer and cleave Spike protein into the S1 and S2 subunits at the furin cleavage site (685-R:S-686), which is also known as a S1/S2 cleavage site. This proteolytic cleavage of the Spike protein by furin results in the separation of the N-terminal S1 subunit, which interacts with hACE2, from the membrane-anchored C-terminal S2 subunit, which is involved in host cell penetration and entry. Docking studies show that furin interacts with surface exposed residues N657–Q690 of SARS-CoV-2 Spike protein (Vankadari, 2020). In particular, residues N657, N658, E661, Y660, T678, N679, S680, R682, R683, R685, S689, and Q690 in the Spike protein form stable contacts with furin (Vankadari, 2020). Remarkably, although cleavage of the S1/S2 site by furin plays a vital role in SARS-CoV-2 infection, it is not sufficient for the membrane fusion process (Papa et al., 2021; Thaingtamtanha and Baeurle, 2022). Upon cleavage by furin, the Spike protein needs to be cleaved at the S2′ site by TMPRSS2 in order to facilitate membrane fusion via exposing FP to the host cell membrane (Papa et al., 2021; Thaingtamtanha and Baeurle, 2022). TMPRSS2 recognizes and binds to the S2′ cleavage site in the SARS-CoV-2 Spike protein, which contains a pair of dibasic motif of “814-KR-815”. This site can be recognized and cleaved by other trypsin-like proteases and is also found in other CoVs (Bestle et al., 2020) although the location and residue composition of this site vary in CoVs. Based on molecular docking, TMPRSS2 and SARS-CoV-2 complex is found to have interactions between the β-sheets of the TMPRSS2’s catalytic domain and the flexible loops of SARS-CoV-2 Spike protein’s cleavage sites (Hussain et al., 2020).

The oral mucosa expresses TMPRSS2 less strongly than the small airway epithelium and nasal epithelium. Thus, the nasal mucosa is the most susceptible site to SARS-CoV-2 infection in the respiratory tract (Liu et al., 2020c). However, if the target cells do not express enough TMPRSS2 or if the Spike protein-hACE2 complex is not accessed by TMPRSS2, SARS-CoV-2 enters host cells by clathrin-mediated endocytosis (Bayati et al., 2021). In this process, CTSL, a lysosomal cysteine protease, plays a vital role by cleaving the Spike protein at two different cleavage sites, T259 (CS-1) and Y636 (CS-2) (Zhao et al., 2022a). Given CTSL’s localization and activity, these cleavage reactions most likely take place in the endosome (Zhao et al., 2022a). CTSL is also known to be involved in SARS-CoV infection (Simmons et al., 2005). Accordingly, out of the seven known human CoVs, only SARS-CoV and SARS-CoV-2 have conserved CTSL cleavage sites at T259 and Y636, indicating that CTSL cleavage is crucial for the infection process of SARS-CoV/SARS-CoV-2 and life cycle (Zhao et al., 2022a). CTSL cleavage sites in Spike protein are highly conserved in SARS-CoV-2 variants including the recent Omicron variant, also supporting the hypothesis that this process is important for the entry of SARS-CoV-2 (Zhao et al., 2022a).

Although furin and TMPRSS2 have been demonstrated to have complementary roles in SARS-CoV-2 infection, some studies show that in the absence of furin, TMPRSS2 and CTSL can still cleave the Spike protein and trigger SARS-CoV-2 infection (Essalmani et al., 2022). Indeed, TMPRSS2 and cathepsins can activate pseudovirus entry while furin alone cannot. Cleavage of Spike protein by furin makes SARS-CoV-2 less reliant on host cells and improves its entry into some target cells, especially those with relatively low TMPRSS2 and/or CTSL expression levels (Shang et al., 2020a). This has also been seen in avian influenza viruses that have been pre-activated with furin (Wrapp et al., 2020).

### 6.2 Effects of mutations on Spike protein cleavage

In the Alpha variant (B.1.1.7), out of the seven missense mutations (N501Y, A570D, P681H, D614G, T716I, S982A, and D1118H) and three deletion mutations (H69, V70, and Y144), the P681H mutation located in the furin cleavage motif is the only one that affects furin cleavage. This mutation in the furin cleavage site, and subsequent conformation change affects the binding affinity of furin to the Spike protein, thereby facilitating viral entry into the host cell and resulting in enhanced viral infectivity (Mohammad et al., 2021). However, no mutations are reported in the cleavage sites of TMPRSS2 and CTSL among all known SARS-CoV-2 variants (Figure 1).

### 6.3 Interventions against viral infection targeting proteolytic cleavage

#### 6.3.1 Furin inhibitors

**FIGURE 6:**
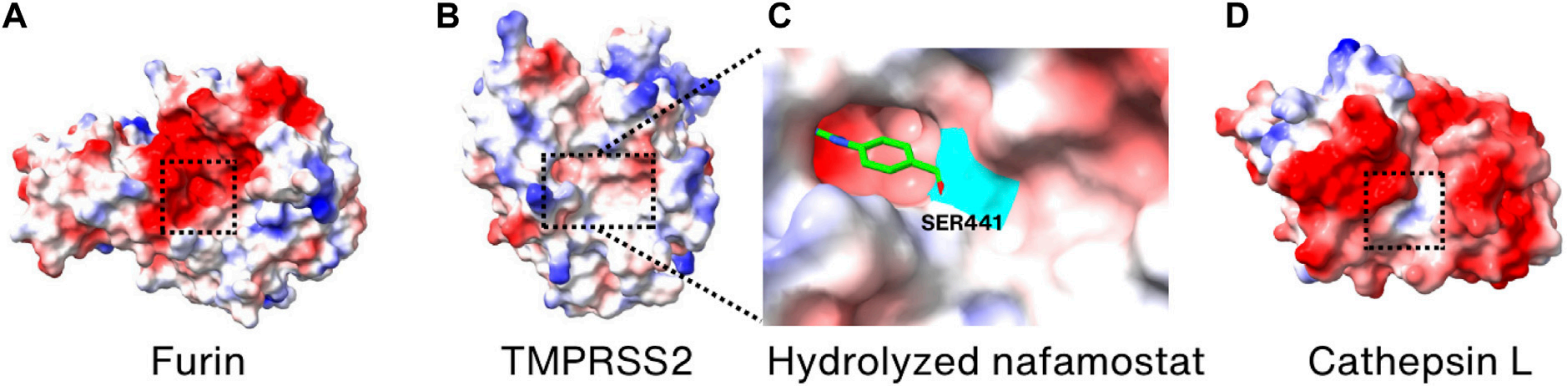
Surface charge distribution models of **(A)** Furin, **(B)** TMPRSS2, **(C)** hydrolyzation product of nafamostat mesylate bound covalently to S441 (highlighted in cyan) of TMPRSS2, and **(D)** cathepsin L. Active sites are marked by the dotted boxes. The crystal structures of Furin, TMPRSS2, TMPRSS2/nafamostat and CTSL (PDB_ID: 6HZD (Van Lam van et al., 2019), 7MEQ (Fraser et al., 2022), 7MEQ (Fraser et al., 2022), and 3K24 (Adams-Cioaba et al., 2011), respectively) were utilized for drawing the models.

Since furin has an important role in promoting SARS-CoV-2 entry into the host cells, furin inhibitors must be potent to prevent SARS-CoV-2 infection. A polybasic stretch of an RRAR motif that matches the consensus sequence of the substrate for furin and related proprotein convertase (PC) family members is present at the S1/S2 boundary only in SARS-CoV-2 (Seidah and Prat, 2012; Coutard et al., 2020). Currently, no FDA-approved drugs specifically target furin for the treatment of COVID-19. While there has been research and interest in furin inhibitors as potential therapies for COVID-19, no clinical trials involving small molecule inhibitors of furin are listed on the online clinical research studies of National Library of Medicine (ClinicalTrials.gov) (Wu et al., 2020a; Villoutreix et al., 2022). The rarity of the application of furin inhibitors may be due to the vital role of furin in maintaining normal physiological functions (Thomas, 2002). Combined with COVID-19 as a disease requiring long-term treatment, inhibition of furin activity through furin inhibitors may result in severe side effects for patients. So far, two furin inhibitors, naphthofluorescein and decanoyl-RVKR-chloromethylketone (CMK), have been known to be effective in preventing infection during the entry stage of SARS-CoV-2 in human cell lines (Cheng et al., 2020). However, their detailed inhibitor mechanism has not yet been elucidated, as no structures of these two inhibitors bound to furin have been determined. Nonetheless, their binding sites and action mechanism seem to be different, since naphthofluorescein is known as a noncompetitive furin inhibitor and CMK is a substrate-mimicking competitive inhibitor that binds to the active site of furin (Cheng et al., 2020) (Figure 6A). Interestingly, Factor Xa, a serine protease, is reported to prevent SARS-CoV-2 infection by cleaving the Spike protein, and thus rivaroxaban, a factor Xa inhibitor, facilitates viral entry (Dong et al., 2023).

#### 6.3.2 TMPRSS2 inhibitors

TMPRSS2 is an enzyme crucial for the entry of SARS-CoV-2 into cells and for viral infection, making it a prime candidate for developing SARS-CoV-2 inhibitors. Nafamostat mesylate and camostat mesylate are known TMPRSS2 inhibitors that have been demonstrated to reduce the infectivity of SARS-CoV-2 in human cell lines (Hoffmann et al., 2020b; Hoffmann et al., 2020c; Mellott et al., 2021). Since both the inhibitors are reactive esters, they are hydrolyzed in the active site of TMPRSS2 to yield a covalently bound phenylguanidino acyl-enzyme complex (Fraser et al., 2022). The crystal structure of inhibitor-enzyme complex reveals that phenylguanidino acyl group is covalently bound to S441 in the active site of TMPRSS2 (Figures 6B, C). However, clinical trials with nafamostat mesylate and camostat mesylate failed in improving the clinical outcomes of COVID-19 hospitalization patients, possibly due to their short plasma half-life (1 h for camostat mesylate and 23.1 min for nafamostat mesylate) and the multiple entry pathways of SARS-CoV-2 during infection (Midgley et al., 1994; Tsukagoshi, 2000; Gunst et al., 2021; Zhuravel et al., 2021; Kinoshita et al., 2022). However, it was reported that nafamostat mesylate significantly decreases the viral load in the nasopharyngeal sample of mild early-onset COVID-19 patients (Okugawa et al., 2023). Considering the structural and functional similarity, the working mechanism of camostat mesylate is expected to be similar to the nafamostat. Therefore, it is anticipated that future efforts in designing TMPRSS2 inhibitors may yield more effective inhibitors that can be used as anti-COVID-19 therapeutics targeting TMPRSS2.

#### 6.3.3 Cathepsin inhibitors

Since CTSL has been demonstrated to have a significant role in the entry of SARS-CoV-2, it has also become one of the targets for developing therapeutics against SARS-CoV-2 (Liu et al., 2020b). Amantadine, known to be a weak antagonist of the NMDA-type glutamate receptor, is used as an anti-influenza drug by preventing uncoating of the virus within the cell (Wharton et al., 1994; Hewitt, 2000). It is also proven to prevent SARS-CoV-2 pseudovirus infection, via reducing the activity of CTSL instead of inhibiting the Spike protein-receptor interaction (Fink et al., 2021; Zhao et al., 2021). Although its exact action mechanism in SARS-CoV-2 is not known, it showed promising results in improving clinical conditions during the phase 3 trials (Rejdak et al., 2022). K777 is also demonstrated to inhibit CTSL *in vitro* and to block the infection of SARS-CoV-2 in various cell lines (Mellott et al., 2021). This inhibitor is in phase 2 clinical trials (Medicine, 2023b). Since it is known to be a cysteine protease inhibitor and covalent inactivator of cathepsins, it is expected to bind to the active site of CTSL (Figure 6D) although its atomic structure with CTSL is not known.

The antiviral effect of CTSL inhibitors is significantly enhanced when combined with TMPRSS2 inhibitors such as nafamostat mesylate (Ashhurst et al., 2021). The combined use of TMPRSS2 inhibitors and CTSL inhibitors can prevent CoV entry into host cells through both endocytic and endosomal pathways. They may be applicable not only in lung epithelial cells but also in other cell types and organs, while simultaneously preserving the adaptive immune system, which may be an effective treatment option for COVID-19 patients (Liu et al., 2020b).

## 7 Membrane fusion step

### 7.1 Conformation of the WT Spike protein after proteolytic cleavage

**FIGURE 7:**
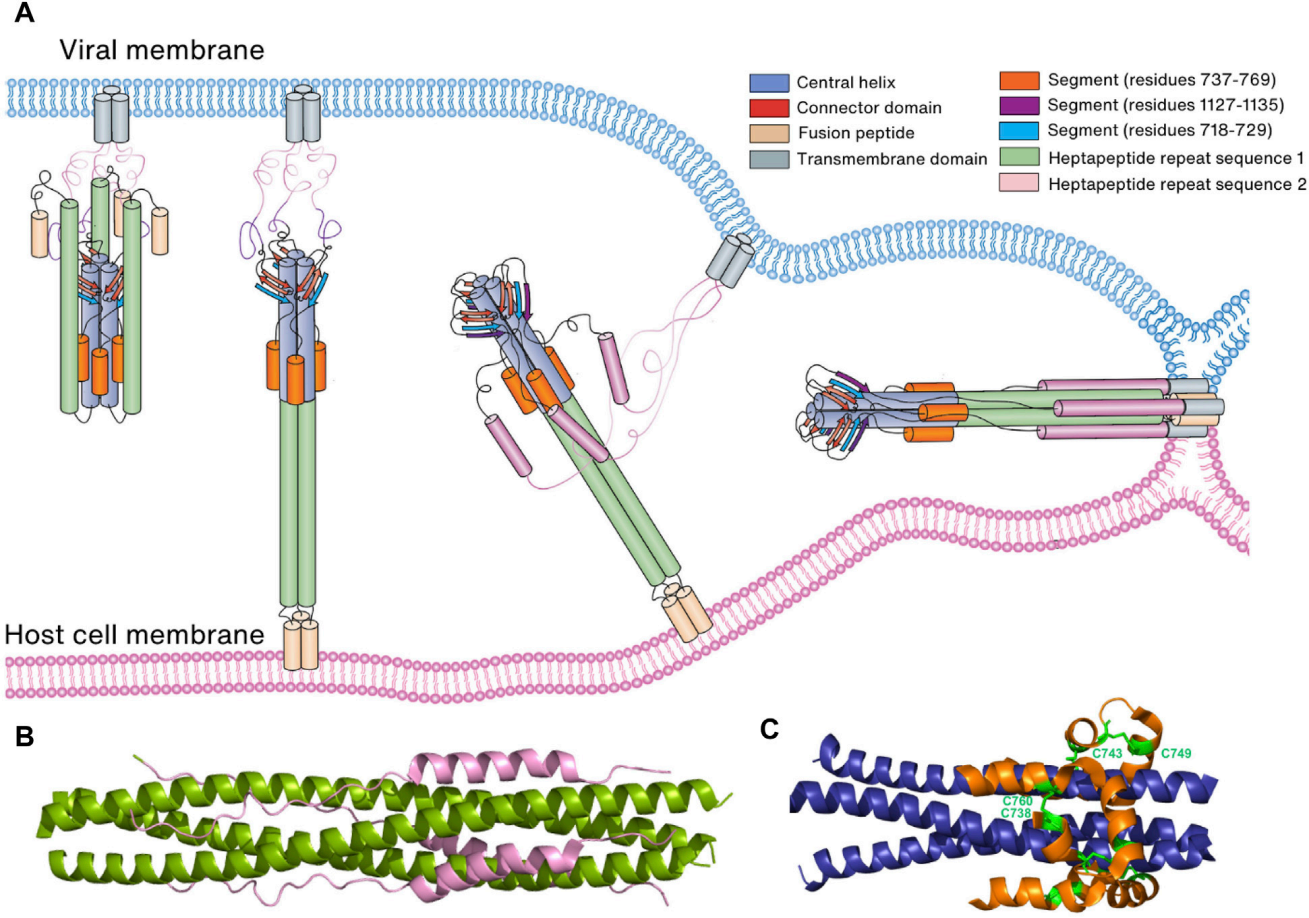
Membrane fusion of the virus to the host cell membrane. **(A)** Schematic representation of membrane fusion. Following cleavage by a host cell protease, the S1 subunit of the Spike protein is eliminated, revealing the S2 subunit. Subsequently, the S2 subunit undergoes conformational changes that bring the two membranes into proximity, leading to the fusion of the virus and host cell membranes. **(B)** Six-helical bundle structure consisting of HR1 (green) and HR2 (pink). **(C)** Six-helical bundle structure consisting of the helices in the N-terminal region of the S2 subunit (orange) and helices in the CH domain (blue). Two disulfide bonds (C738:C760 and C743:749) stabilize the bundle structure.

After proteolytic cleavage by TMPRSS2 and CTSL, the S1 subunit is dissociated from the S2 subunit, exposing the FP located on the C-terminal side of S2′ in the S2 subunit. Interestingly, the N-terminal region of the S2 subunit, encompassing residues 686–815, folds into a strand and a helix. Although it is separated from the rest of the S2 subunit after cleavage at the S2′ site, it still interacts with other regions of the subunit. With the release of steric constraints by the S1 subunit and the loosening of packing between HR1 helices and trimeric CH helices, HR1 extends freely from the helical bundle consisting of HR1, CH, and helices present in the N-terminal region of the S2 subunit, which allows FP, HR1, and CH to form a continuous long three-helical bundle, enabling FP to reach the host cell membrane (Figure 7A) (Walls et al., 2016; Bestle et al., 2020). In the prebinding step, HR2 is disordered but folds into a helix and tightly packs against the three-helical bundle formed by HR1, which results in the formation of a stable 6-HB structure (Xia et al., 2020b; Cai et al., 2020) (Figure 7B). MD studies suggest that HR2 initially forms a stable three-helical bundle before attaching to HR1, acting as a zipper alongside CH and HR1 long helix to form 6-HB with HR1 (Dodero-Rojas et al., 2021). Eventually, the formation of a 6-HB brings the viral membrane into contact with the host cell membrane. This contact is facilitated by the HR2 and TM domains, followed by the embedding of the FP (Dodero-Rojas et al., 2021) (Figure 7A).

In both prebinding and postfusion conformations, an invariant three-stranded β-sheet is observed. This β-sheet comprises two strands originating from the CD domain and one strand (residues 718–729) derived from the N-terminal region of the S2 subunit (Cai et al., 2020; Shi et al., 2023). However, in the postfusion conformation, an additional segment (residues 1,127–1,135) joins this arrangement to create a four-stranded β-sheet structure. This structural rearrangement is thought to play a pivotal role in extending HR2 towards the viral cell membrane and initiating the folding of HR2 (Cai et al., 2020; Shi et al., 2023). Furthermore, a six-helical bundle comprising helices from a segment (residues 737–769) in the N-terminal region of the S2 subunit and helices from CH remains conserved in both prebinding and postfusion conformations. This structural stability is reinforced by two disulfide bonds (C738:C760 and C743:C749) (Cai et al., 2020; Shi et al., 2023) (Figure 7C). Collectively, these findings suggest that the three-stranded β-sheet and the six-helical bundle serve as anchors, retaining their conformations and interactions during the prefusion-to-postfusion transition. This stability facilitates conformational changes in other regions of the Spike protein, including HR1 and HR2.

### 7.2 Effects of mutations on membrane-fusion conformation

S982A and D1118H mutations in the Alpha variant and T1027I and V1176F mutations in the Gamma variant in the S2 subunit do not trigger conformational change, but D950N located in the HR1 domain of the Delta variant is reported to promote the membrane fusion of SARS-CoV-2, especially when combined with the P681R mutation (Furusawa et al., 2023). Omicron variant contains four mutations in the S2 subunit: N856K, Q954H, N969K, and L981F. While Q954H, N969K, and L981F mutations affect the overall architecture of the HR1-HR2, their interaction is marginal. In contrast, the N856K mutation causes a dramatic decrease in the fusogenicity of the Omicron variant. Experimental results also show a decrease in viral infection when introducing the N856K mutation, but an increase when restoring N856 (Sun et al., 2023). This result can be explained by the formation of a salt bridge between K856 and D586, which stabilizes the FP proximal region (residues 828–853) (Zhang et al., 2022; Sun et al., 2023).

### 7.3 Interventions against viral infection targeting the postfusion conformation

In the S2 subunit of Spike protein, HR1, HR2, and the C-terminal domain play a significant role in the formation of postfusion conformation and in membrane fusion. These domains combine to form 6-HB-like assemblies, which results in membrane fusion necessary for viral entrance (Cai et al., 2020; Zhang et al., 2021a). Since the assembly of the 6-HB bundle in the Spike protein’s S2 subunit is believed to provide a protective function for SARS-CoV-2, targeting the HR domain might inhibit its refolding. This refolding is essential for blocking membrane fusion and limiting infection (Outlaw et al., 2020; Wang et al., 2020). In addition, HR1-targeting-peptide inhibitors also inhibit HR1-HR2 formation, thereby preventing virus entry and membrane fusion (Xia et al., 2020b). EK1C4 peptides, a fusion inhibitor from pan-coronavirus targeting the HR1 domain of S2, have been shown to inhibit HR1-HR2 formation by binding to HR1(Xia et al., 2020b). HR2-sequence-based peptides (IPB02, HR2P) were designed to bind with the HR1 domain, thus preventing the formation of HR1 and HR2, ultimately stopping membrane fusion (Xia et al., 2020c; Zhu et al., 2020). Salvianolic acid C, a compound isolated from a natural herb, was also found to inhibit the assembly of the 6-HB fusion core by interacting with residues S940, T941, A942, L945, K947, L948, and Q949 in the SARS-CoV-2 HR1 groove (Yang et al., 2020). In addition to the HR1-HR2 interaction, the highly conserved HR2 motif (residues 1,145–1,178) and its upstream linker loop (residues 1,105–1,143) are also crucial for HR1-HR2 formation and can be a potential target for the development of broad-spectrum vaccine candidates and therapeutics against coronaviruses in the near future (Fan et al., 2020). FP-targeted antibodies such as COV44-62 and COV44-79 prevent FP binding to the host cell membrane and virus entry (Dacon et al., 2022). Stem-helix-specific antibodies inhibit the formation of 6-HB and thus prevent the fusion of the two membranes (Pinto et al., 2021). As of now, multiple stem helix-targeted nAbs have been isolated from recovering COVID-19 patients (S2P6, CC40.8, CV3-25, hr2.016, CC25.106, CC95.108, CC68.109, CC99.103, and CC95.102) or immunization-induced mice (WS6, B6, 1.6C7, 28D9, IgG22, and G5) (Wang et al., 2021a; Li et al., 2021b; Hsieh et al., 2021; Pinto et al., 2021; Sauer et al., 2021; Hurlburt et al., 2022; Shi et al., 2022; Zhou et al., 2022; Bianchini et al., 2023; Zhou et al., 2023). Antibodies against additional S2 subunit components have also been shown to prevent viral entry mediated by Spike protein (Lip et al., 2006; Coughlin and Prabhakar, 2012).

**FIGURE 8:**
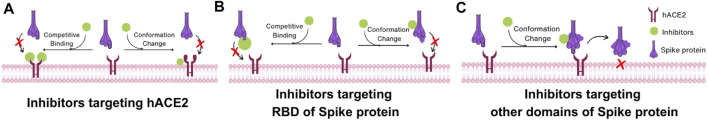
Interaction mechanism of SARS-CoV-2 inhibitors based on Spike protein structure. **(A)** Inhibitors targeting hACE2. Inhibitors in this category impede the interaction between hACE2 and the RBD of the Spike protein by two mechanisms: competing with the RBD of the Spike protein or inducing a conformational change in hACE2 that reduces its binding affinity to the RBD. **(B)** Inhibitors targeting RBD of Spike protein. These inhibitors hinder the interaction between the hACE2 and the RBD through two mechanisms: competing with hACE2 for binding to the RBD or inducing a conformational change in the RBD of Spike protein. **(C)** Inhibitors targeting other domains of Spike protein. These inhibitors alter the conformation of the Spike protein, which makes the Spike protein unable to change its structure for membrane fusion.

Based on the structural understanding of the mechanism of SARS-CoV-2 uptake by host cells, COVID-19 therapeutics can be classified into three broad classes: inhibitors targeting hACE2, inhibitors targeting RBD of Spike protein, and inhibitors targeting other regions of the Spike protein other than the RBD. The first class of inhibitors binds to hACE2, disrupting the interaction between hACE2 and the RBD of the Spike protein. These inhibitors either target hACE2-RBD binding via competitive inhibition of the binding site or by inducing a conformational change in hACE2 that reduces its affinity for RBD binding (Figure 8A). For instance, dalbavancin binds to hACE2, inhibiting SARS-CoV-2 entry into cells via competitive inhibition. The second class of inhibitors targets the RBD of Spike protein and thence can also inhibit the interaction between hACE2 and RBD either by competing with hACE2 for binding or inducing a conformational change of RBD (Figure 8B). For example, antibodies targeting the RBM compete with hACE2 binding while antibodies targeting the non-RBM region of the RBD still inhibit binding to hACE2 possibly via induction of a conformational change. In addition to the RBD, the third class of inhibitors targeting other domains of the Spike protein may also be developed to induce changes in conformation when these inhibitors bind to Spike protein domains and inhibit viral binding (Figure 8C). This is the mechanism of action of nAbs targeting NTD.

## 8 Conclusion and future perspectives

The entry of SARS-CoV-2 into the host cell requires many steps in which Spike proteins play a pivotal role. During the entry process, the Spike protein, comprising of the S1 and S2 subunits, undergoes structural changes at every stage. The conformational change and cleavage of the Spike protein during this process is necessary for adequate entry of SARS-CoV-2 into host cells, and thus understanding their roles and conformational change in each step is required for the development of effective prevention strategies. Accordingly, many chemicals, peptides, and antibodies targeting Spike proteins and their associated proteins including the host cell receptors and proteases are being developed. In terms of developing small molecule inhibitors, drug repurposing combined with computer-based approaches such as machine-learning, molecular docking, and MD simulation led to the development and identification of many useful compounds for the treatment of COVID-19. Although clinical trials of TMPRSS2 inhibitors nafamostat mesylate and camostat mesylate for COVID-19 treatment failed, drug identification by repurposing remains a promising approach. For example, the TMPRSS2 inhibitor BC-11, recently identified by this approach, has the potential to be further optimized for clinical trials to treat COVID-19 since it shows several advantages in terms of drug development due to its small molecular weight (Tsukagoshi, 2000; Moumbock et al., 2023).

New approaches such as nanobodies and aptamers targeting RBD and preventing RBD-hACE2 interactions also provide new opportunities for COVID-19 treatments (Liu et al., 2021c; Sun et al., 2021; Xu et al., 2021; Yang et al., 2022a). Nanobodies represent a single variable domain of heavy-chain-only antibody that can be synthesized *in vitro* or derived from the immune systems of llamas (Jovčevska and Muyldermans, 2020; Bao et al., 2021). Due to their smaller size, better penetration, and more homogeneous distribution than traditional antibodies, nanobodies are more favorable than antibodies for accessing target epitopes during SARS-CoV-2 entry. In addition, nucleic acid-based aptamers have been demonstrated to outperform antibodies since they are more stable, have a longer half-life, are more heat-resistant, show no immune response, and are simpler to produce than antibody. In addition, no animals or cell cultures are required for the production of aptamers (Thiviyanathan and Gorenstein, 2012). Therefore, nanobodies and aptamers will be expected to be alternatives for traditional antiviral therapeutics.

Remarkably, most Omicron subvariants can evade the majority of nAbs (Cox et al., 2023). This characteristic presents challenges in the continued discovery and fabrication of antibodies. mAbs development may focus on conserved regions of SARS-CoV-2 variants or identify mutation epitopes that affect SARS-CoV-2 resistance. The recent successful identification of post-cleavage or postfusion conformation provides further understanding of the structures and mechanisms of conformation changes, and also new targets to develop inhibitors, vaccines, and nAbs to prevent SARS-CoV-2 entry into host cells (Shi et al., 2023). The high number of Spike protein structures of SARS-CoV-2, as well as the influence of its mutations, creates opportunities to develop therapeutics against coronaviruses.
